# Seroprevalence of human herpesvirus-8 (HHV-8) in countries of Southeast Asia compared to the USA, the Caribbean and Africa

**DOI:** 10.1038/sj.bjc.6690782

**Published:** 1999-11

**Authors:** D Ablashi, L Chatlynne, H Cooper, D Thomas, M Yadav, A W Norhanom, A K Chandana, V Churdboonchart, S A R Kulpradist, M Patnaik, K Liegmann, R Masood, M Reitz, F Cleghorn, A Manns, P H Levine, C Rabkin, R Biggar, F Jensen, P Gill, N Jack, J Edwards, J Whitman, C Boshoff

**Affiliations:** Advanced Biotechnologies Inc, 9108 Guilford Road, Columbia, MD 21046, USA; Pusat Asasi Sains, University of Malaya, Kuala Lumpur 50603, Malaysia; Department of Pathology, Mahidol University, Rama VI Road-Phayathai, Bangkok 10400, Thailand; Specialty Laboratories Inc., 2211 Michigan Avenue, Santa Monica, CA 90404, USA; Division of Hematology, School of Medicine, University of Southern California, 1441 Eastlake Avenue, Los Angeles, CA 90033, USA; Institute of Human Virology, University of Maryland, 725 W. Lombard Street, Baltimore, MD 21201, USA; Viral Epidemiology Branch, National Cancer Institute, 6130 Executive Boulevard, EPN/434, Bethesda, MD 20852, USA; Epidemiology Department, George Washington University, School of Medicine, Ross Hall, 2300 I Street NW, Washington, DC 20037-2337, USA; Immune Response Corporation, 5935 Darwin Court, Carlsbad, CA 92008, USA; Medical Research Foundation of Trinidad and Tobago, Port of Spain, Trinidad; Chester Beatty Laboratories, Institute of Cancer Research, 237 Fulham Road, London SW3 6JB, UK

## Abstract

Seroprevalence of HHV-8 has been studied in Malaysia, India, Sri Lanka, Thailand, Trinidad, Jamaica and the USA, in both healthy individuals and those infected with HIV. Seroprevalence was found to be low in these countries in both the healthy and the HIV-infected populations. This correlates with the fact that hardly any AIDS-related Kaposi’s sarcoma has been reported in these countries. In contrast, the African countries of Ghana, Uganda and Zambia showed high seroprevalences in both healthy and HIV-infected populations. This suggests that human herpes virus-8 (HHV-8) may be either a recently introduced virus or one that has extremely low infectivity. Nasopharyngeal and oral carcinoma patients from Malaysia, Hong Kong and Sri Lanka who have very high EBV titres show that only 3/82 (3.7%) have antibody to HHV-8, demonstrating that there is little, if any, cross-reactivity between antibodies to these two gamma viruses. © 1999 Cancer Research Campaign


*British Journal of Cancer* (1999) **81**(5), 893–897
© 1999 Cancer Research Campaign
Article no. bjoc.1999.0782

### Seroprevalence of human herpesvirus-8 (HHV-8) in countries of Southeast Asia compared to the USA, the Caribbean and Africa

**D Ablashi[1], L Chatlynne[1], H Cooper[1], D Thomas[1], M Yadav[2], AW Norhanom[2], AK Chandana[2], V Churdboonchart[3], SAR Kulpradist[3], M Patnaik[4], K Liegmann[4], R Masood[5], M Reitz[6], F Cleghorn[6], A Manns[7], PH Levine[8], C Rabkin[7], R Biggar[7], F Jensen[9], P Gill[5], N Jack[10], J Edwards[10], J Whitman[1] and C Boshoff[11]**

[1]Advanced Biotechnologies Inc, 9108 Guilford Road, Columbia, MD 21046, USA; [2]Pusat Asasi Sains, University of Malaya, Kuala Lumpur 50603, Malaysia; [3]Department of Pathology, Mahidol University, Rama VI Road-Phayathai, Bangkok 10400, Thailand; [4]Specialty Laboratories, Inc., 2211 Michigan Avenue, Santa Monica, CA 90404, USA; [5]Division of Hematology, School of Medicine, University of Southern California, 1441 Eastlake Avenue, Los Angeles, CA 90033, USA; [6]Institute of Human Virology, University of Maryland, 725 W. Lombard Street, Baltimore, MD 21201, USA; [7]Viral Epidemiology Branch, National Cancer Institute, 6130 Executive Boulevard, EPN/434, Bethesda, MD 20852, USA; [8]Epidemiology Department, George Washington University, School of Medicine, Ross Hall, 2300 I Street NW, Washington, DC 20037-2337, USA; [9]Immune Response Corporation, 5935 Darwin Court, Carlsbad, CA 92008, USA; [10]Medical Research Foundation of Trinidad and Tobago, Port of Spain, Trinidad; [11]Chester Beatty Laboratories, Institute of Cancer Research, 237 Fulham Road, London SW36JB, UK

**Summary** Seroprevalence of HHV-8 has been studied in Malaysia, India, Sri Lanka, Thailand, Trinidad, Jamaica and the USA, in both healthy individuals and those infected with HIV. Seroprevalence was found to be low in these countries in both the healthy and the HIV-infected populations. This correlates with the fact that hardly any AIDS-related Kaposi's sarcoma has been reported in these countries. In contrast, the African countries of Ghana, Uganda and Zambia showed high seroprevalences in both healthy and HIV-infected populations. This suggests that human herpes virus-8 (HHV-8) may be either a recently introduced virus or one that has extremely low infectivity. Nasopharyngeal and oral carcinoma patients from Malaysia, Hong Kong and Sri Lanka who have very high EBV titres show that only 3/82 (3.7%) have antibody to HHV-8, demonstrating that there is little, if any, cross-reactivity between antibodies to these two gamma viruses. © 1999 Cancer Research Campaign

Human herpesvirus-8 (HHV-8), also known as Kaposi's sarcoma associated herpesvirus (KSHV), is associated with the aetiopathogenesis of Kaposi's sarcoma (KS), body cavity B-cell lymphoma, also called primary effusion lymphoma (PEL), and multicentric Castleman's disease (MCD) (Chang et al, 1994; Chang and Moore, 1996; Ganem, 1996; IARC, 1997; Boshoff and Weiss, 1998; Schulz, 1998). Current serological and molecular assays indicate that HHV-8 infection is not widespread, but predominantly restricted to populations at risk of AIDS-associated, endemic, or classic KS (Chang and Moore, 1996; IARC, 1997; Boshoff and Weiss, 1998; Schulz, 1998). The assays employed to measure HHV-8 antibodies include immunofluorescence assays (IFA) for latent and lytic proteins (Gao et al, 1996*a*; Kedes et al, 1996; Miller et al, 1996); ELISA for latent associated nuclear antigen (LANA) (Oskenhendler et al, 1998; Gao et al, 1996*b*), ORF 65 (Simpson et al, 1996), ORF 26 (Davis et al, 1997), and whole virus (Chatlynne et al, 1998); as well as K8.1 (Raab et al, 1998), capsid protein (Lin et al, 1997), and BC-1 cell extract (Schulz, 1998) immunoblots.

There are no serological data available on the prevalence of HHV-8 in Southeast Asia or the Indian subcontinent. Although HIV is now a common problem in this subcontinent (Bhoopat et al, 1994), classic and endemic KS do not occur (Wabinga et al, 1993; Ziegler, 1993; Bhoopat et al, 1994; Sukpanichant et al, 1998), and the incidence of AIDS-associated KS appears very low (Bhoopat et al, 1994; Sukpanichant et al, 1998). We investigated the prevalence in healthy individuals of HHV-8 (Table 1) in Southeast Asia ($n$ = 234 from Malaysia and Thailand), Indian subcontinent ($n$ = 188 from India and Sri Lanka), the West Indies ($n$ = 410 from Jamaica and Trinidad), and three African countries ($n$ = 164 from Uganda, Zambia and Ghana) and compared this with healthy donors' sera from the USA ($n$ = 135). Where available, we also tested a limited number of sera from HIV-1-infected patients (Table 2) for HHV-8 antibody to see if the prevalence differed from the healthy population: India ($n$ = 42), Thailand ($n$ = 196), Zambia ($n$ = 25), Uganda ($n$ = 35), Trinidad ($n$ = 12) and the USA ($n$ = 124).

*Received 4 January 1999*
*Revised 29 March 1999*
*Accepted 5 April 1999*

*Correspondence to:* DV Ablashi

### MATERIALS AND METHODS

We employed a recently described whole virus enzyme-linked immunosorbent assay (ELISA; Advanced Biotechnologies Inc, Columbia, MD, USA) using sucrose-banded HHV-8 virus lysate as the source of antigen (Chatlynne et al, 1998). A whole virus ELISA has advantages over using individual viral proteins as a source of antigen, since the viral lysate contains a broad spectrum of seroreactivity compared to that detected by a single viral protein in ELISA, IFA, or immunoblots. Since this ELISA detects predominantly proteins to lytic antigens, we also compared this ELISA to a lytic antigen IFA in a subset of KS- and HIV-positive

**Table 1** Detection of IgG antibody to HHV-8 (KSHV) lytic proteins by ELISA of populations from the USA, India, Sri Lanka, Thailand, Malaysia, Jamaica, Trinidad, Uganda, Zambia and Ghana

| Country | Age (years) range | Male–Female | Group | Number of antibody positive/number tested[a] | Per cent positive |
|---|---|---|---|---|---|
| Infected controls | 54–92 | 19–1 | A. Classical (HIV-negative) | 20/20 | 100.0 |
| (Kaposi's sarcoma) | 33–61 | 10–0 | B. HIV-infected | 9/10 | 90.0 |
| 1. *USA* | 22–78 | 87–48 | Blood donors | 7/135 | 5.2 |
| 2. *Southeast Asia* | | | | | |
| India[b] | 20–58 | 56–52 | Healthy individuals | 4/108 | 4.0 |
| Thailand[c] | 17–69 | 39–36 | Healthy individuals | 3/75 | 4.0 |
| Malaysia | 9–85 | 113–46 | Healthy individuals | 7/159 | 4.4 |
| Sri Lanka[b] | 21–78 | 48–32 | Healthy individuals | 3/80 | 3.8 |
| 3. *Caribbean* | | | | | |
| Jamaica | 18–64 | 124–123 | Blood donors | 9/250 | 3.6 |
| Trinidad[d] | 20–>50 | 89–67 | Blood donors | 2/160 | 1.3 |
| 4. *Africa* | | | | | |
| Uganda | 18–67 | 44–18 | Healthy individuals | 24/62 | 38.7 |
| Zambia | 17–68 | 22–18 | Healthy individuals | 15/40 | 37.5 |
| Ghana | 13–72 | 47–15 | Healthy individuals | 26/62 | 41.9 |

[a]All sera were tested for IgG at a dilution of 1:80 or 1:100 using sucrose density gradient purified HHV-8 as source of antigen. Negative: OD ≤ 0.113, equivocal: OD > 0.113 and < 0.128, positive: OD ≥ 0.128. HHV-8 antibody positive sera were rerun for conformation by IFA for lytic antigens at dilutions of 1:40. A few antibody negative sera from each country were run as controls. [b]Sera were collected in order to screen for HHV-6 and EBV. [c]Sera were collected to screen for HIV. Age and sex of three individuals were not traceable. [d]Four individuals' age and sex data were not available.

**Table 2** Distribution of HHV-8 IgG antibody in HIV-1-positive individuals who do not have Kaposi's sarcoma[a]

| Country | Group | Age (years) range | Male–Female | No. of antibody positive/No. tested | Per cent Positive |
|---|---|---|---|---|---|
| USA | HIV + homosexual men | 21–55 | 62–0 | 27/62 | 43.5 |
| | HIV + Non-KS (current)[b] | 33–52 | 16–0 | 8/16 | 50.0 |
| | HIV + Non-KS (retrospective)[c] | 31–58 | 46–0 | 13/46 | 28.3 |
| India | HIV-1 + | 20–58 | 30–12 | 1/42 | 2.4 |
| No HIV-associated KS reported | | | | | |
| Thailand | HIV-1+ | 19–41 | 100–96 | 22/196 | 11.2 |
| No HIV-associated KS reported | | | | | |
| Uganda | HIV-1+ | 20–62 | 22–13 | 16/35 | 45.7 |
| Zambia | HIV-1+ | 22–69 | 18–7 | 11/25 | 44.0 |
| Trinidad | HIV-1+ | 20–50 | 9–3 | 0/12 | 0 |

[a]Sera were tested by ELISA to purified virus, some LNA (BCP-1 cell antibody), and by IFA to lytic proteins (KS-1 cell substrate). [b]Six of the eight that were antibody positive have since developed KS. [c]Patients either died or did not develop KS in the 10 years following the sample.

patient sera ($n = 58$). For the ELISA, sera were diluted at least 1:80 and for lytic IFA, sera were tested at a dilution of 1:40. Frozen and coded sera were shipped to the laboratory in Columbia, Maryland, for testing. Serum (obtained from Malaysia, Hong Kong and Sri Lanka) from patients with nasopharyngeal carcinoma, oral carcinoma and other diseases (infectious mononucleosis and African Burkitt's lymphoma) were also titred for Epstein–Barr virus (EBV) to see if patients with high EBV titres also tested positive for HHV-8, and to see if antibodies to EBV might cross-react with HHV-8 (Table 3).

### Collection of serological samples

### *Serum/plasma samples from the USA*

The healthy subjects consisted of blood donors and laboratory workers. The samples were obtained from a blood bank and drawn in a clinical laboratory. The HIV+, classical KS, and HIV+ KS sera came from the Department of Dermatology, New York University Medical Center, North Shore University Hospital (kindly provided by Drs A Friedman-Kien and Mark Kaplan), and the Norris Cancer Research Center, University of Southern California, Los Angeles (collected by Dr P Gill).

### *Samples from India*

These were collected from individuals at blood bank facilities in Bombay, Madras and New Delhi for HIV testing. Therefore, the samples consisted of both healthy donors (HIV-negative) and HIV-positive individuals who were antibody-positive by radioimmunoassay and Western blot.

### *Samples from nasopharyngeal carcinoma, oral carcinoma, infectious mononucleosis, African Burkitt's lymphoma and from healthy donors and HIV+ patients from Africa*

Most of the nasopharyngeal carcinoma (NPC) and all of the oral carcinoma (OC) sera were provided by the University of Malaya, Kuala Lumpur. All of the OC sera originally came from Sri Lanka

 © 1999 Cancer Research Campaign

**Table 3** Sera with high EBV antibody titres have no cross-reactivity with HHV-8

| Sera type | No. tested | Male–Female | Age (years) range | Range of EBV-VCA antibody titres[a] | Range of EBV-EA antibody titres[b] | EBV IgG (titre) of HHV-8 antibody positive samples | HHV-8 IgG antibody (%) | (titre) |
|---|---|---|---|---|---|---|---|---|
| Nasopharyngeal carcinoma patients (Malaysia and Hong Kong) | 42 | 32–10 | 35–78 | IgG 1:640–>1:5120 | IgA 1:80–>1:320 | 1:1280 1:2560 | 2/42 (4.8) | 1:80 1:160 |
| Oral squamous carcinoma patients (Sri Lanka) | 40 | 30–10 | 21–82 | IgG 1:640–≥1:1280 | ND[c] | 1:640 | 1/40 (2.5) | ≥1:80 |
| Infectious mononucleosis patients (USA) | 7 | 5–2 | 17–25 | IgM 1:40–≥1:160 | ND | – | 0/7 (0) | |
| Burkitt's lymphoma children (Ghana) | 12 | 8–4 | 6–16 | IgG 1:320–≥1:1280 | IgG 1:80–1:320 | 1:640 | 1/12 (8.3) | (1:160) |

[a]The sera for antibody to EBV-VCA were tested by IFA using P3HR-1 Clone 1B cells of which 20% expressed VCA. [b]The antibody to EBV-EA was tested by IFA on Raji cells superinfected with EBV isolated from P3HR-1 cells. At 48 hours post infection, >50% of the Raji cells expressed EA. [c]ND – not done to the University of Malaya for HHV-6/HHV-7 and other studies. The other NPC, African Burkitt's lymphoma (BL), and infectious mononucleosis (IM) sera were obtained from the Department of Microbiology/Immunology, Georgetown University School of Medicine, Washington, DC. These NPC sera originally came from Hong Kong (kindly supplied by Dr Ng). The BL sera (Table 3) from Ghana were originally part of another study on EBV conducted by Drs Robert Bigger and Paul Levine (National Cancer Institute's (NCI) Burkitt's lymphoma project). These sera were collected in the late 1960s and stored at the NCI Natural Products Repository. Some of the African sera (Uganda and Zambia) were made available by the Georgetown University, Washington, DC, the Cancer Research Institute, London, UK and the Natural Products Repository, National Cancer Institute, Frederick Cancer Research and Development Center, Frederick, Maryland. All of the healthy donors' sera from Ghana were also obtained from the Natural Products Repository of NCI and were collected in 1990 and 1991 from the Roforidua region for other NCI studies (ID = Ghana/Africa, ID = ward controls and ID = Kocle hospital).

*Serum samples from Malaysia*

These were collected from healthy donors by two of the authors, Drs Yadav and Norhanom, at the Institute of Advanced Studies, University of Malaya, as a part of an EBV seroepidemiology study. These sera were obtained from Chinese, Malays and Indians, whereas the NPC sera were from Chinese and Malays. These sera were stored at the University facility at –20°C.

*Serum samples from Thailand*

Serum samples of HIV-negative donors were collected at the Mahidol University of Thailand. The serum samples from HIV-positive donors were collected from the Remune Trail Center. Among the healthy donors (HIV-negative), three were found to be HHV-8 antibody-positive workers who applied for a permit to seek work in the Middle East. The age and sex of four serum samples were not traceable (Table 1).

*Serum samples from Jamaica*

Sera samples from normal blood donors were obtained from the National Transfusion Service, Kingston, Jamaica.

*Serum samples from Trinidad*

All samples from Trinidad were collected (Drs Cleghorn, Jack and Edwards) after informed consent from adult STD clinic attendees as part of a larger NIH-funded study to screen for early HIV-1 infection in the period 1995–1997.

### Age and sex of donors

Age and sex of individuals from whom sera were collected varied in each group, whether collected at a clinic (as previously indicated), blood bank, or as part of a study group from healthy adults in the USA, India, Sri Lanka, Malaysia, Thailand, Uganda, Zambia, Ghana, Jamaica and Trinidad, and HIV-infected individuals from the USA, India, Trinidad, Thailand, Uganda, Zambia and Ghana (with and without KS). The age (range) and sex distribution of all sera tested are provided in Table 1, 2 and 3.

### RESULTS AND DISCUSSION

Table 1 shows the distribution of HHV-8 IgG antibodies in healthy individuals from the countries tested. We used sera from patients with KS for a positive control, and all of these sera were positive for HHV-8 IgG antibody. The prevalence of HHV-8 in 5.2% of blood donors in the USA correlates with that previously reported using both latent IFA and immunoblot assays (Gao et al, 1996*a*; Kedes et al, 1996; Miller et al, 1996). The higher prevalence of HHV-8 in adults from Uganda (38.7%) and Zambia (37.5%) correlates with the incidence of endemic KS and the high incidence of AIDS-associated KS in these countries (Wabinga et al, 1993). In Ghana, however, the high seroprevalence of HHV-8 (41.9%) does not correlate with an increased incidence of African endemic KS (Ziegler, 1993), indicating that co-factors are probably necessary to precipitate endemic KS (Ziegler, 1993; Bourboulia et al, 1998). The high seroprevalence of HHV-8 antibody to lytic viral antigens further supports another study of pregnant African women and prostitutes from Cameroon (Bestetti et al, 1998) in which 52.6% who were HIV-positive demonstrated IgG antibody to HHV-8, compared to 42.6% who were HIV-negative. This is the first report to document the prevalence of KSHV in the Far East in healthy individuals (Table 1) and in HIV-1-positive individuals from these

*© 1999 Cancer Research Campaign* 

countries (Table 2). As in other countries where endemic and classic KS are rare (e.g. Northern America and North Western Europe), HHV-8 infection appears restricted to less than 5% of healthy adults. Similarly, HHV-8 is not a common infection of healthy adults in the two West Indian countries tested: Trinidad (1.3%) and Jamaica (3.6%) (Manns et al, 1998). In HIV-1-infected populations, seroprevalence of HHV-8 is generally elevated above that of the general population in areas where KS is associated with HIV infection (e.g. USA); but in areas (India, Thailand) where HIV infection is on the increase, the lack of evidence of KS does not correlate. In spite of the high prevalency of HIV and AIDS in India and Thailand, the seroprevalency of HHV-8 is much lower in this population than in comparable HIV-positive populations in the USA or Africa (Table 2). The number of HIV-positive samples from Trinidad is too small to draw any conclusions; however, it may be similar to India and Thailand.

Since infection with EBV is widespread in the Far East and Indian subcontinent and is associated with NPC and endemic Burkitt's lymphoma, we tested sera from 40 patients with NPC from Malaysia and Hong Kong, from 40 patients with oral squamous carcinoma (OC) from Sri Lanka, from seven IM patients in the USA, and from 12 children with BL from Ghana to determine whether there is any immunologic cross-reactivity between the two human gamma herpesviruses, i.e. EBV and HHV-8, using this ELISA (Table 3). EBV antibodies were tested by IFA for anti-VCA IgG using a preparation of P3HR-1 cells (Clone 1B), that is about 20% positive for EBV viral capsid antigen (VCA). IgG and IgA antibody to EBV early antigen (EA) was tested by IFA with Raji cells superinfected with the P3HR-1 strain of EBV. After 48 h superinfection, approximately 60% of the cells are positive for EA as determined by monoclonal antibodies to EA-diffuse (EA-D) and EA-restricted (EA-R) (kindly provided by Dr Gary Pearson of the Georgetown University School of Medicine, Washington, DC). To test for EBV IgM, sera were first preabsorbed to remove IgG antibodies and then incubated on fixed P3HR-1 cells for 3 h. Table 3 shows the results of this study. The 42 NPC patients had very high IgG and IgA antibody titres to EBV-VCA and EBV-EA, but only two of these patients demonstrated antibodies to HHV-8. In addition, one serum from 40 patients with OC tested positive for HHV-8 IgG. Out of 12 BL patients with antibody to EBV, one had antibody to HHV-8. Seven IM sera with EBV IgM antibody were all negative for HHV-8 IgG. The titres of HHV-8 IgG-positive sera, i.e. NPC, OC and BL, are given in Table 3, which showed that the titres were not elevated (as observed in KS sera) (Chatlynne et al, 1998) in spite of the high EBV titres antibody (Table 3). This supports a lack of cross-reactivity between HHV-8 and anti-EBV antibodies in this ELISA, and further indicates that the HHV-8 IgG antibody detected in the general populations, HIV-positive individuals, and KS patients of the various countries tested was specific. This data further supports our previous finding where KS sera, after adsorption for EBV antibody, retained the HHV-8 IgG (Chatlynne et al, 1998).

In this study, we show that HHV-8 is not a common human pathogen in Southeast Asia or the Caribbean. Current data indicate that HHV-8 infection is more prevalent in countries where classic and endemic KS occurs (Whitby et al, 1998). In these countries, horizontal transmission and mother-to-child transmission are likely to occur (Bourboulia et al, 1998; Luppi et al, 1998; Gessain et al, 1999). In countries with a low incidence of classic or endemic KS, HHV-8 is predominantly found in homosexual men at risk of developing KS (Luppi et al, 1998; Martin et al, 1998; Verbeek et al, 1998), and is a sexually transmitted agent among these individuals (Martin et al, 1998). In Southeast Asia and the Caribbean, HHV-8 might be a relatively newly introduced pathogen. The restricted distribution of HHV-8 in these populations also correlates with the relatively low incidence of reported cases of classical KS or AIDS KS (Gill, personal communication, 1998; Jensen, personal communication, 1998). Longitudinal follow-up studies of individuals infected with HHV-8 in Southeast Asia and the Caribbean will help to assess the participation of this viral agent in the pathogenesis of KS and other diseases or malignancies. Future studies will attempt to determine the mode(s) of transmission of HHV-8 in the Far East and whether HHV-8 was present in sera collected prior to the AIDS epidemic.

### ACKNOWLEDGEMENT

We thank Ms Ruth Weinroth for editorial assistance in the preparation of this paper.

### REFERENCES

Bestetti G, Renon G, Mauclere P, Ruffie A, Mbopi Keou FX, Emo D, Parravicini C, Corbellino M, de Thé G and Gessain A (1998) High seroprevalence of human herpesvirus-8 in pregnant women and prostitutes from Cameroon. *AIDS* **12**: 541–543

Bhoopat L, Thampraser K, Chalwan B, Attasiri C, Vithayasal P and Chaimongkoi B (1994) Histopathologic spectrum of AIDS associated lesions in Maharaj Nakon Chaing Mai Hospital. *Asian Pac J Immunol* **12**: 95–104

Boshoff C and Weiss R (1998) Kaposi's sarcoma-associated herpesvirus. *Adv Cancer Biol Res* **75**: 57–86

Bourboulia D, Whitby D, Boshoff C, Newton R, Beral V, Carrara H, Lane A and Sitas F (1998) Serological evidence for vertical transmission of KSHV/HHV-8 in healthy South African children. *J Am Med Assoc* **280**: 31–32

Chang Y and Moore PS (1996) Kaposi's sarcoma (KS)-associated herpesvirus and its role in KS. *Infect Agents Dis* **5**: 215–222

Chang Y, Cesarman E, Pessin MS, Lee F, Culpepper J, Knowles DM and Moore PS (1994) Identification of herpes-like DNA sequences in AIDS-associated Kaposi's sarcoma. *Science* **265**: 1865–1869

Chatlynne LG, Lapps W, Handy M, Huang YQ, Masood R, Hamilton AS, Said JW, Koeffler HP, Kaplan MH, Friedman-Kien A, Gill PS, Whitman JE and Ablashi DV (1998) Detection and titration of human herpesvirus-8-specific antibodies in sera from blood donors, acquired immunodeficiency syndrome patients, and Kaposi's sarcoma patients using a whole virus enzyme-linked immunosorbent assay. *Blood* **92**: 53–58

Davis DA, Humphrey RW, Newcomb FM, O'Brien TR, Goedert JJ, Strauss SF and Yarchoan R (1997) Detection of serum antibodies to Kaposi's sarcoma-associated herpesvirus specific peptides. *J Infect Dis* **175**: 1971–1979

Ganem D (1996) Human herpesvirus 8 and the biology of Kaposi's sarcoma. *Semin Virol* **7**: 325–332

Gao SJ, Kingsley L, Li M, Zheng W, Parravinci C, Zeigler J, Newton R, Rinaldo CR, Saah A, Phair J, Detels R, Chang Y and Moore P (1996*a*) KSHV antibodies among Americans, Italians, and Ugandans with and without Kaposi's sarcoma. *Nat Med* **2**: 925–928

Gao SJ, Kingsley L, Hoover DR, Spira TJ, Rinaldo CR, Saah A, Phair J, Detels R, Parry P, Chang Y and Moore PS (1996*b*) Seroconversion to antibodies against Kaposi's sarcoma-associated herpesvirus-related latent nuclear antigens before the development of Kaposi's sarcoma. *N Engl J Med* **335**: 233–241

Gessain A, Mauclère P, Van Beveren M, Plancoulaine S, Ayouba A, Essame-Oyono J-L, Martin PMV and de Thé G (1999) Human herpesvirus-8 primary infection occurs during childhood in Cameroon, Central Africa. *Intl J Cancer* **81**: 189–192

Gill PS (1998) Personal Communication. University of Southern California, Los Angeles, CA, USA

IARC Monographs Volume 70 (1997) On Epstein-Barr Virus and Human Herpesvirus-8. Published by World Health Organization Intl Agency for Res on Cancer, 1997

Jensen F (1998) Personal Communication. Immune Response Corporation, Carlsbad, CA, USA

 © 1999 Cancer Research Campaign

Kedes DH, Operskalski E, Busch M, Kohn R, Flood J and Ganem D (1996) The seroepidemiology of human herpesvirus-8 (Kaposi's sarcoma-associated herpesvirus): distribution of infection in KS risk groups and evidence for sexual transmission. *Nat Med* **2**: 918–924

Lin SF, Sun R, Heston L, Granville L, Shedd D, Haglaund K, Rigsby M and Miller G (1997) Identification, expression, and immunogenicity of Kaposi's sarcoma-associated herpesvirus-encoded small viral capsid antigen. *J Virol* **71**: 3069–3076

Luppi MD, Whitby D, Barozzi P, Boshoff C, Weiss RW, Cucci F and Torelli G (1998) Age- and sex-specific seroprevalence of human herpesvirus 8 in Jamaica – response. *J Natl Cancer Inst* **90**: 1103–1104

Manns A, Strickler HD, Hanchard B, Manassaram DM, Waters D and Ablashi DV (1998) Age- and sex-specific seroprevalence of human herpesvirus-8 in Jamaica. *J Natl Cancer Inst* **90**: 1102–1103

Martin JN, Ganem DE, Osmond DH, Page-Schafer KA, Macrae D and Kedes DH (1998) Sexual transmission and the natural history of human herpesvirus-8 infection. *N Engl J Medicine* **338**: 948–954

Miller G, Rigsby MO, Heston L, Grogan E, Sun R, Metroka C, Levy JA, Gao SJ, Chan Y and Moore P (1996) Antibodies to butyrate-inducible antigens of Kaposi's sarcoma-associated herpesvirus in patients with HIV-1 infection. *N Engl J Med* **334**: 1292–1297

Oskenhendler E, Cazals-Hatem D, Schulz TF, Barateau V, Grollet L, Sheldon J, Clauvel JP, Sigaux F and Agbalika F (1998) Transient angiolymphoid hyperplasia and Kaposi's sarcoma after primary infection with human herpesvirus-8 in a patient with human immunodeficiency virus infection. *N Engl J Med* **338**: 1585–1590

Raab MS, Albrecht JC, Birkman A, Yaguboglu S, Lang D, Fleckenstein B and Neipel F (1998) The immunogenic glycoprotein gp35–37 of human herpesvirus-8 is encoded by open reading frame K8.1. *J Virol* **72**: 6725–6731

Schulz TF (1998) Kaposi's sarcoma-associated herpesvirus (human herpesvirus-8). *J Gen Virol* **79**: 1573–1591

Simpson GR, Schulz TF, Whitby D, Cook PM, Boshoff C, Rainbow L, Howard MR, Gao SJ, Bohenzky RA, Simmonds P, Lee C, deRuiter A, Hatzakism A, Tedder RS, Weller IVD, Weiss RA and Moore PS (1996) Prevalence of Kaposi's sarcoma associated herpesvirus infection measured by antibodies to recombinant capsid protein and latent immunofluorescence antigen. *Lancet* **348**: 1133–1138

Sukpanichant S, Sonakul D, Plankijagum A, Wanachiwanawin W, Veerakul G, Mahasandana C, Tanphalchitr VS and Suvatte V (1998) Malignant lymphoma in Thailand. *Cancer* **83**: 1197–1204

Verbeek W, Frankel M, Miles S, Said J and Koeffler HP (1998) Seroprevalence of HHV-8 antibodies in HIV-positive homosexual men without Kaposi's sarcoma and their clinical follow-up. *Microbiol Infect Dis* **109**: 778–783

Wabinga HR, Pakin DM, Wabwire-Mangen F and Mugerwa JW (1993) Cancer in Kampala, Uganda in 1989–1991: changes in incidence in the era of AIDS. *Intl J Cancer* **54**: 23–36

Whitby D, Luppi M, Barozzi P, Boshoff C, Weiss RA and Torelli G (1998) HHV-8 seroprevalence in blood donors and lymphoma patients from different regions of Italy. *J Natl Cancer Inst* **90**: 395–397

Ziegler JL (1993) Endemic Kaposi's sarcoma in Africa and local volcanic soils. *Lancet* **342**: 1348–1351

*© 1999 Cancer Research Campaign*

## References

[PDF_00375] Bestetti G., Renon G., Mauclére P., Ruffié A., Mbopi Kéou F. X., Eme D., Parravicini C., Corbellino M., de Thé G., Gessain A. (1998). High seroprevalence of human herpesvirus-8 in pregnant women and prostitutes from Cameroon.. AIDS.

[PDF_00379] Bhoopat L., Thamprasert K., Chaiwun B., Attasiri C., Vithayasai P., Chaimongkol B., Limpichankit T., Sirisanthana V. (1994). Histopathologic spectrum of AIDS-associated lesions in Maharaj Nakorn Chiang Mai Hospital.. Asian Pac J Allergy Immunol.

[PDF_00382] Boshoff C., Weiss R. A. (1998). Kaposi's sarcoma-associated herpesvirus.. Adv Cancer Res.

[PDF_00384] Bourboulia D., Whitby D., Boshoff C., Newton R., Beral V., Carrara H., Lane A., Sitas F. (1998). Serologic evidence for mother-to-child transmission of Kaposi sarcoma-associated herpesvirus infection.. JAMA.

[PDF_00389] Chang Y., Cesarman E., Pessin M. S., Lee F., Culpepper J., Knowles D. M., Moore P. S. (1994). Identification of herpesvirus-like DNA sequences in AIDS-associated Kaposi's sarcoma.. Science.

[PDF_00387] Chang Y., Moore P. S. (1996). Kaposi's Sarcoma (KS)-associated herpesvirus and its role in KS.. Infect Agents Dis.

[PDF_00392] Chatlynne L. G., Lapps W., Handy M., Huang Y. Q., Masood R., Hamilton A. S., Said J. W., Koeffler H. P., Kaplan M. H., Friedman-Kien A. (1998). Detection and titration of human herpesvirus-8-specific antibodies in sera from blood donors, acquired immunodeficiency syndrome patients, and Kaposi's sarcoma patients using a whole virus enzyme-linked immunosorbent assay.. Blood.

[PDF_00407] Gao S. J., Kingsley L., Hoover D. R., Spira T. J., Rinaldo C. R., Saah A., Phair J., Detels R., Parry P., Chang Y. (1996). Seroconversion to antibodies against Kaposi's sarcoma-associated herpesvirus-related latent nuclear antigens before the development of Kaposi's sarcoma.. N Engl J Med.

[PDF_00403] Gao S. J., Kingsley L., Li M., Zheng W., Parravicini C., Ziegler J., Newton R., Rinaldo C. R., Saah A., Phair J. (1996). KSHV antibodies among Americans, Italians and Ugandans with and without Kaposi's sarcoma.. Nat Med.

[PDF_00411] Gessain A., Mauclère P., van Beveren M., Plancoulaine S., Ayouba A., Essame-Oyono J. L., Martin P. M., de Thé G. (1999). Human herpesvirus 8 primary infection occurs during childhood in Cameroon, Central Africa.. Int J Cancer.

[PDF_00424] Kedes D. H., Operskalski E., Busch M., Kohn R., Flood J., Ganem D. (1996). The seroepidemiology of human herpesvirus 8 (Kaposi's sarcoma-associated herpesvirus): distribution of infection in KS risk groups and evidence for sexual transmission.. Nat Med.

[PDF_00428] Lin S. F., Sun R., Heston L., Gradoville L., Shedd D., Haglund K., Rigsby M., Miller G. (1997). Identification, expression, and immunogenicity of Kaposi's sarcoma-associated herpesvirus-encoded small viral capsid antigen.. J Virol.

[PDF_00435] Manns A., Strickler H. D., Hanchard B., Manassaram D. M., Waters D., Ablashi D. V. (1998). Age- and sex-specific seroprevalence of human herpesvirus 8 in Jamaica.. J Natl Cancer Inst.

[PDF_00438] Martin J. N., Ganem D. E., Osmond D. H., Page-Shafer K. A., Macrae D., Kedes D. H. (1998). Sexual transmission and the natural history of human herpesvirus 8 infection.. N Engl J Med.

[PDF_00441] Miller G., Rigsby M. O., Heston L., Grogan E., Sun R., Metroka C., Levy J. A., Gao S. J., Chang Y., Moore P. (1996). Antibodies to butyrate-inducible antigens of Kaposi's sarcoma-associated herpesvirus in patients with HIV-1 infection.. N Engl J Med.

[PDF_00445] Oksenhendler E., Cazals-Hatem D., Schulz T. F., Barateau V., Grollet L., Sheldon J., Clauvel J. P., Sigaux F., Agbalika F. (1998). Transient angiolymphoid hyperplasia and Kaposi's sarcoma after primary infection with human herpesvirus 8 in a patient with human immunodeficiency virus infection.. N Engl J Med.

[PDF_00450] Raab M. S., Albrecht J. C., Birkmann A., Yağuboğlu S., Lang D., Fleckenstein B., Neipel F. (1998). The immunogenic glycoprotein gp35-37 of human herpesvirus 8 is encoded by open reading frame K8.1.. J Virol.

[PDF_00453] Schulz T. F. (1998). Kaposi's sarcoma-associated herpesvirus (human herpesvirus-8).. J Gen Virol.

[PDF_00455] Simpson G. R., Schulz T. F., Whitby D., Cook P. M., Boshoff C., Rainbow L., Howard M. R., Gao S. J., Bohenzky R. A., Simmonds P. (1996). Prevalence of Kaposi's sarcoma associated herpesvirus infection measured by antibodies to recombinant capsid protein and latent immunofluorescence antigen.. Lancet.

[PDF_00461] Sukpanichnant S., Sonakul D., Piankijagum A., Wanachiwanawin W., Veerakul G., Mahasandana C., Tanphaichitr V. S., Suvatte V. (1998). Malignant lymphoma in Thailand: changes in the frequency of malignant lymphoma determined from a histopathologic and immunophenotypic analysis of 425 cases at Siriraj Hospital.. Cancer.

[PDF_00464] Verbeek W., Frankel M., Miles S., Said J., Koeffler H. P. (1998). Seroprevalence of HHV-8 antibodies in HIV-positive homosexual men without Kaposi's sarcoma and their clinical follow-up.. Am J Clin Pathol.

[PDF_00467] Wabinga H. R., Parkin D. M., Wabwire-Mangen F., Mugerwa J. W. (1993). Cancer in Kampala, Uganda, in 1989-91: changes in incidence in the era of AIDS.. Int J Cancer.

[PDF_00470] Whitby D., Luppi M., Barozzi P., Boshoff C., Weiss R. A., Torelli G. (1998). Human herpesvirus 8 seroprevalence in blood donors and lymphoma patients from different regions of Italy.. J Natl Cancer Inst.

[PDF_00473] Ziegler J. L. (1993). Endemic Kaposi's sarcoma in Africa and local volcanic soils.. Lancet.

